# Programming and Reprogramming the Viscoelasticity and Magnetic Response of Magnetoactive Thermoplastic Elastomers

**DOI:** 10.3390/polym15234607

**Published:** 2023-12-03

**Authors:** Sergei A. Kostrov, Josiah H. Marshall, Mitchell Maw, Sergei S. Sheiko, Elena Yu. Kramarenko

**Affiliations:** 1 Faculty of Physics, Lomonosov Moscow State University, Leninskie Gory 1/2, 119991 Moscow, Russia; kostrov@polly.phys.msu.ru; 2 Department of Chemistry, University of North Carolina at Chapel Hill, Chapel Hill, NC 27599, USA; jmarshall@unc.edu (J.H.M.); mitrmaw5@live.unc.edu (M.M.)

**Keywords:** magnetoactive elastomer, magnetorheological effect, molecular bottlebrushes

## Abstract

We present a novel type of magnetorheological material that allows one to restructure the magnetic particles inside the finished composite, tuning in situ the viscoelasticity and magnetic response of the material in a wide range using temperature and an applied magnetic field. The polymer medium is an A-g-B bottlebrush graft copolymer with side chains of two types: polydimethylsiloxane and polystyrene. At room temperature, the brush-like architecture provides the tissue mimetic softness and strain stiffening of the elastomeric matrix, which is formed through the aggregation of polystyrene side chains into aggregates that play the role of physical cross-links. The aggregates partially dissociate and the matrix softens at elevated temperatures, allowing for the effective rearrangement of magnetic particles by applying a magnetic field in the desired direction. Magnetoactive thermoplastic elastomers (MATEs) based on A-g-B bottlebrush graft copolymers with different amounts of aggregating side chains filled with different amounts of carbonyl iron microparticles were prepared. The in situ restructuring of magnetic particles in MATEs was shown to significantly alter their viscoelasticity and magnetic response. In particular, the induced anisotropy led to an order-of-magnitude enhancement of the magnetorheological properties of the composites.

## 1. Introduction

Magnetoactive elastomers (MAEs) [1,2,3,4,5,6,7,8,9,10] are composite materials consisting of magnetic nano- or microparticles embedded into the polymer matrix. Under the influence of an external magnetic field, these materials can change their physical properties due to magnetic interactions of magnetic particles (MPs) and magnetoelastic coupling. MAEs can deform [11,12,13,14,15,16] and change electric [17,18,19,20] and rheological [21,22,23,24,25,26,27,28,29] properties. An enormous magnetic response is used as the basis for many of the important practical applications of the MAEs, in particular, in soft robotics, vibration control and haptic feedback devices, active braking systems, pressure and magnetic field sensors, etc. [30,31,32,33,34,35,36,37,38]. Some of the possible applications are reviewed in [39].

It is well known that the softer the MAE, the higher its magnetorheological response [4]. For example, in [40], an extremely soft MAE with a storage modulus of less than 1 kPa exhibited an increase in its value by four orders of magnitude when placed in a 300 mT magnetic field. However, making polymer matrices this soft is a challenging task due to the entanglement of polymer chains. At low exposure time (or high frequency), such topological restraints act as effective cross-links. The traditional approach to overcome this limitation is to use low-molecular-weight oil as a plasticizer [41,42]. Materials produced with this method are susceptible to oil leakage, which can occur during deformation or over time. Recently, we reported a different approach to the design of supersoft MAEs based on polymer networks with brush-like strands and demonstrated that they achieve the same level of magnetic response as highly diluted traditional linear MAEs but without the addition of a low-molecular-weight plasticizer [43]. The elimination of hazardous leakage components, while maintaining the softness and tissue mimetic properties of the matrix, expands the applications of these materials, particularly in medicine.

The development of magnetopolymer materials with an oriented distribution of MPs is another promising area of research. A traditional way to fabricate an anisotropic material is by curing material in a magnetic field [18,22,44,45,46,47,48,49,50,51,52,53,54,55,56,57,58]. Anisotropic MAEs have a number of properties that differ from their isotropic counterparts [59,60,61,62,63,64,65,66]. It is clear that many physical properties of anisotropic materials are direction-dependent. For example, an anisotropic material can be a conductor in the direction of MP alignment, while the isotropic counterpart is an insulator [18]. Magnetorheological properties also change significantly: Anisotropic MAEs tend to be stiffer and have a larger absolute modulus gain in the magnetic field due to the better interaction of MPs in chain-like structures [22,23,24,53,54,55]. The anisotropic distribution of MPs inside the material is one of the main ways to fabricate soft robots based on MAEs [30,46,47,48,49].

In a recent work [67], we proposed a new platform for the design of magnetoactive materials whose properties can be programmed after curing. The developed magnetoactive composite was based on a thermoplastic matrix formed by bottlebrush graft copolymers (A-g-B) with PS and PIB side chains. The side chains effectively dilute the system, thus providing us with the tissue mimetic softness of the material without using any additional plasticizer and strain stiffening. In addition, the PS side chains form network nodes because they are incompatible with the PIB side chains at room temperature. These nodes melt reversibly at moderate temperatures, which opens up the possibility of the in situ restructuring of MPs using both temperature and magnetic field. We call these materials magnetoactive thermoplastic elastomers (MATEs) to distinguish them from traditional chemically cross-linked MAEs and to emphasize the thermoactivity of the matrix. In MAEs, the MPs can only be restructured once during material synthesis, and after synthesis, the MPs are fixed in position, so that shifts in the magnetic field are reversible. In MATEs, on the other hand, one can melt the material at any time, set the desired distribution of MPs using an external magnetic field, and then cool the material back to room temperature (RT), thus “freezing” the MPs in a new configuration. Note that due to the non-chemical nature of the polymer network, it is not necessary to go above the melting temperature to restructure the MPs.

In this work, we present a novel MATE based on an A-g-B bottlebrush graft copolymer with side chains of two types: polydimethylsiloxane and polystyrene. We experimentally study the magnetorheological properties of MATEs with 5, 10, and 20 vol% of carbonyl iron as MPs (*φ_MP_*) based on a polymer bottlebrush with different architecture (3% and 4% of aggregating PS block *φ_A_*), show the possibility to restructure MPs inside the polymer matrix in situ, and investigate the impact of such restructuring. Thus, we demonstrate the universality of the proposed A-g-B platform and show for the first time the reprogramming of material properties by applying a magnetic field in different directions.

## 2. Materials and Methods

**Figure 1:**
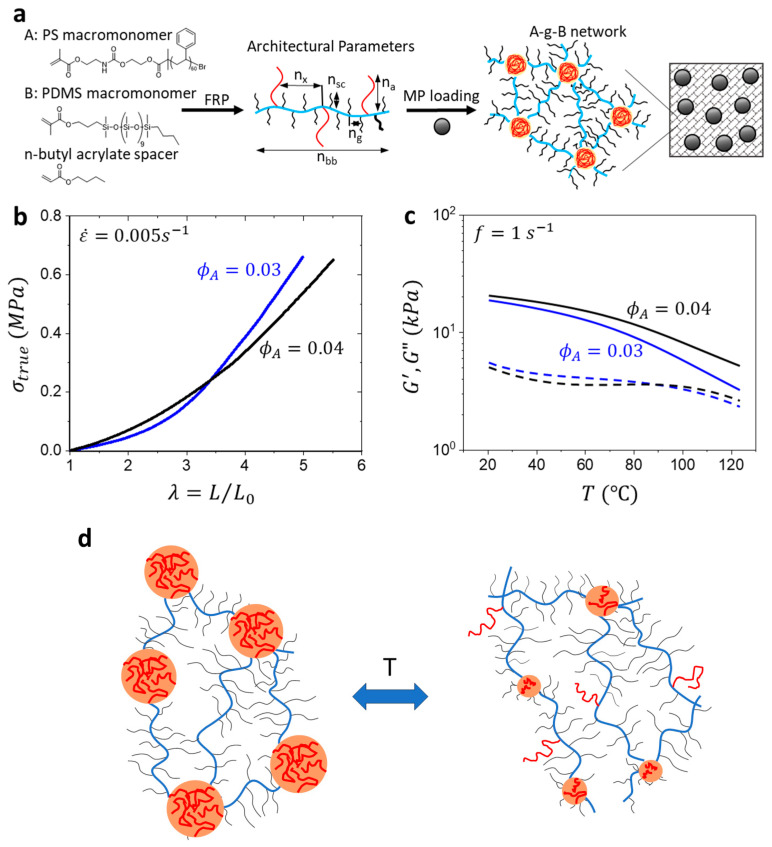
Preparation of A-g-B MATEs: (**a**) chemical structure and the architectural parameters of A-g-B copolymer; a schematic representation of A-g-B matrix filled with magnetic microparticles; (**b**) tensile stress–strain curves and (**c**) temperature dependence of the storage modulus, $G^{\prime }$, and the loss modulus, $G^{\prime \prime }$, of A-g-B elastomers with different volume fractions of the PS block as indicated at the frequency *ω* = 10 rad/s; (**d**) schematic representation of temperature changes in the matrix structure.

The viscoelastic properties of A-g-B bottlebrush graft polymers are defined by a unique set of architectural parameters that include the degree of the polymerization (DP) of the brush backbone (n_bb_), the DP between side chain units (n_g_), the DP of the brush side chain (n_sc_), the DP between the A-block units (n_x_), and the DP of the A-block units (n_A_) [68]. The total volume fraction of the A-block monomer incorporated in the network is expressed as *φ_A_*. A-g-B synthesis proceeded via free radical copolymerization of the A and B-block macromonomers, polydimethysiloxane (PDMS), and polystyrene (PS) (Figure 1a). PDMS was sourced commercially from Gelest Inc., Morrisville, PA, USA^®^, whereas the PS was fabricated with atom transfer radical polymerization (ATRP) using a hydroxyl-terminated α-haloketone initiator, which was functionalized post-polymerization using 2-methyl isocyanoethyl methacryate. N-butyl acrylate (nBA) was used as a spacer molecule to control n_g_. PDMS, PS, and nBA were copolymerized over a 24 h period under an inert atmosphere, and the crude mixture was subsequently washed to remove unreacted monomer. ^1^H NMR was used to determine the n_x_ and n_g_ of the system (Figures S1 and S2).

### 2.1. Preparation of Magnetic Composites

Two A-g-B copolymer compositions with different volume fractions (*φ_A_* = 0.03 and 0.04) of polystyrene A-block (n_A_ = 60) and respective n_x_ values were prepared. Both A-g-B systems were blended with magnetic iron microparticles (ρ = 7.8 g/cm^3^) at a volume fraction of *φ_MP_* = 0.05, 0.10, and 0.20. The MPs used in this study were spherical magnetically soft carbonyl iron particles of 4.5 μm in diameter (R-20 brand from Spektr-Khim, Moscow, Russia). MAEs were prepared by vortex mixing and sonication of the A-g-B polymer with ~10 mass equivalents of dichloromethane and the calculated mass of particles before transferring the blend to a Teflon Petri dish for the flash evaporation (110 °C) of the solvent. The dry MAE samples were then hot-melt-pressed at 85 °C into a 1 mm thick homogenous film to prepare them for analysis.

### 2.2. Mechanical Characterization of A-g-B: Tensile and T-Sweep

Due to the microphase separation of the PS and PDMS blocks, the synthesized polymers form thermoplastic elastomers with a characteristic stress–strain J-curve when subjected to a uniaxial tensile measurement at a strain rate of $\dot{\varepsilon }=0.005\;s^{-1}$ (Figure 1b). An RSA-G2 DMA (TA Instruments) was used for uniaxial tensile measurements. Samples were procured using a steel die dog bone cutter with dimensions 12 mm (length) × 2 mm (width).

Using an Anton Paar Physica MCR 302 rheometer with 20 mm disk geometry, the neat materials were subjected to a temperature sweep (20–120 °C) at a constant frequency (10 rad/s) and strain (0.1%). Upon heating, A-g-B elastomers soften to allow for particle rearrangement in a magnetic field (Figure 1c). Matrix softening is expected to result from a reduction in the incompatibility between A and B units as temperature increases. This leads to a change in the micellar structure, similar to ordinary AB copolymers, and a partial breakdown of the physical network (schematically shown in Figure 1d). Notably, $G^{\prime }>G^{\prime \prime }$ within the temperature range examined.

The magnetorheological study was performed with an Anton Paar Physica MCR 302 (Anton Paar, Graz, Austria) rheometer equipped with an MRD 170/1T magnetic cell. The magnetic cell has an electromagnet capable of generating a homogeneous magnetic field perpendicular to the measuring plate. The magnetic flux density B can be varied from zero to 1T by varying the drive current in the coil of the electromagnet in the range 0–5 A. The lower plate of the measuring unit was stationary. The movement of the upper plate was set to perform harmonic oscillations γ = γ_0_ sin(ωt), where γ is the shear strain, γ_0_ is a shear strain amplitude, and ω is the angular frequency. Disk-shaped samples with a diameter of 20 mm were cut from the film and placed between the plates of the measuring system. The height of all samples was about 1 mm. For all tests, we set low shear strain amplitude γ_0_ = 0.1%, which corresponds to the linear viscoelastic response of the material. For the frequency test, the angular frequency was varied in the range ω = 1–100 rad/s. In other measurements, the angular frequency was assumed to be 10 rad/s unless otherwise stated.

## 3. Results

### 3.1. Magnetic Response at Room Temperature

**Figure 2:**
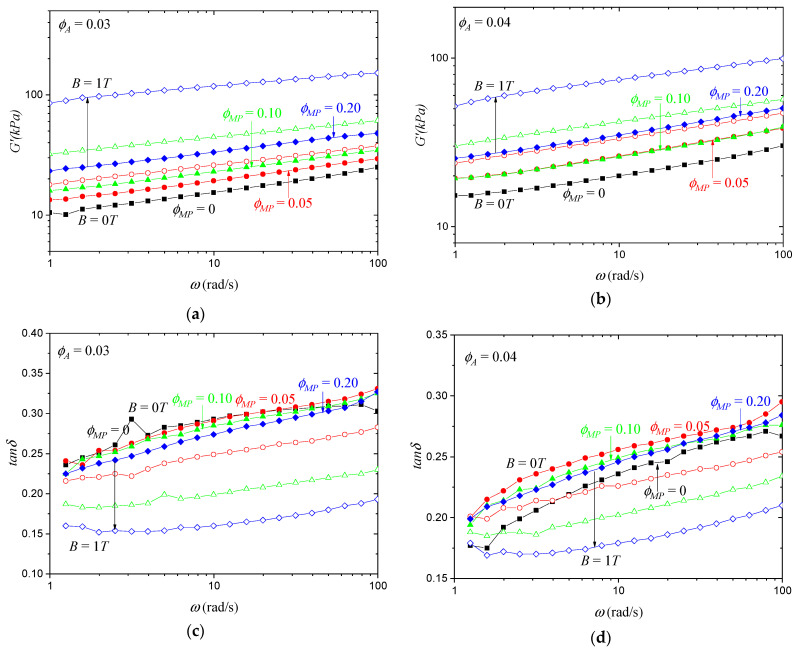
Frequency dependence of (**a**,**b**) storage modulus and (**c**,**d**) damping factor of MATEs based on polymer with (**a**,**c**) 3% PS side chains and (**b**,**d**) 4% PS chains with different concentrations of MPs as indicated, measured at magnetic field values of B = 0T (solid symbols) and B = 1T (empty symbols). MPs volume fraction *φ_MP_* = 0 (black squares) 0.05 (red circles) 0.10 (green triangles) and 0.20 (blue diamonds). Strain amplitude dependences of the storage modulus and damping factor are presented in Figure S3.

It is well known that magnetically active elastomers can be significantly reinforced when exposed to a magnetic field. Figure 2 shows the frequency dependence of the storage modulus and damping factor of the synthesized isotropic MATEs measured at magnetic field values of B = 0T and B = 1T. All samples exhibited the so-called magnetorheological effect: an increase in the elastic modulus in the magnetic field. This effect was more pronounced at higher concentrations of MPs. The sample with *φ_A_* = 0.03 and *φ_MP_* = 0.20 showed an increase of the storage modulus in the magnetic field by 257% from 33 to 118 kPa at an angular frequency ω = 10 rad/s.

The damping factor, $\tan\delta =G^{\prime \prime }/G^{\prime }$, indicates the ratio of dissipated energy to elastically stored energy during one cycle of mechanical loading. In the magnetic field, the damping factor decreased (Figure 2c,d), which also indicates the reinforcement of the MATEs.

### 3.2. Orientation of MPs Inside MATEs

**Figure 3:**
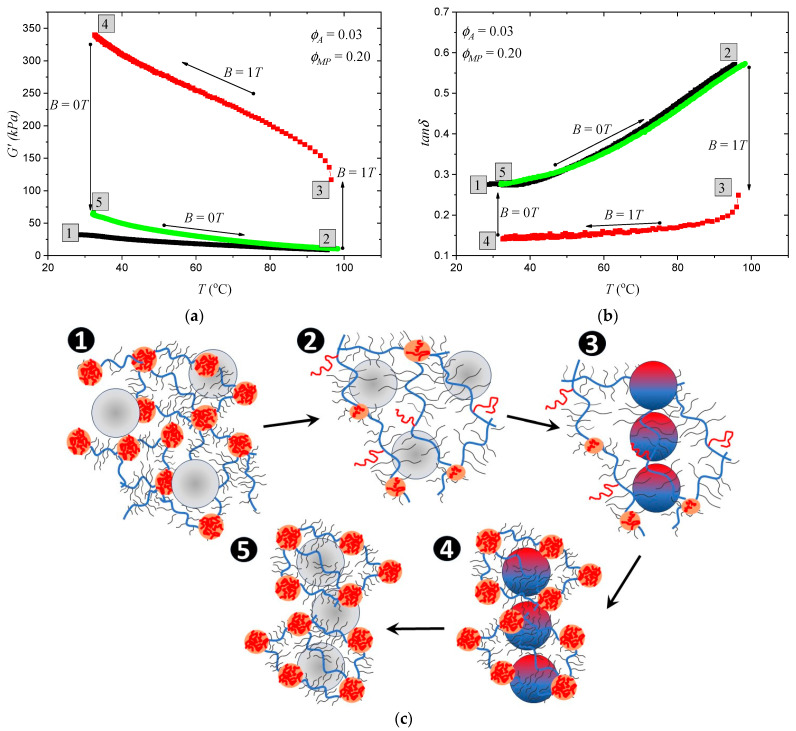

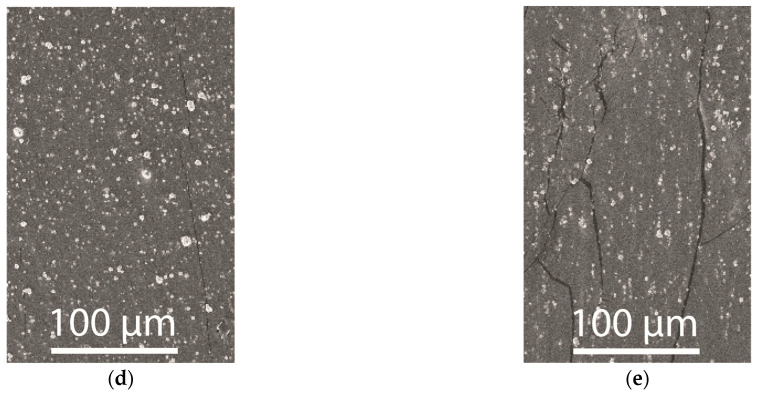
Temperature dependence of (**a**) storage modulus and (**b**) damping factor of MATE based on polymer matrix with 3% of PS containing 20 vol% of MPs. Arrows indicate the direction of the temperature change. The sample was heated to soften the polymer matrix (black line). After that, the magnetic field was applied, and the temperature returned to room temperature (red line) to fix MPs in chain-like ordered positions. Green line shows the next cycle of sample heating. The corresponding dependences for the other samples are presented in Figures S4 and S5; (**c**) schematic image of the polymer state and MP distribution at corresponding points of the curves (**a**,**b**). SEM images of MATE’s cross-section in (**d**) point 1 and (**e**) point 5.

The developed MATEs open the possibility of creating anisotropic structures of magnetic particle distribution in situ by using both magnetic field and temperature. Figure 3a,b show the process step by step. Point 1 corresponds to the initial state of the sample: The polymer matrix was stiff, and MPs were randomly distributed inside the matrix. Then, the sample was heated, and at point 2, the polymer matrix became softer, which led to a decrease in the storage modulus of the material and an increase in the damping factor. Although the damping factor did not exceed unity, the “living” polymer matrix still allowed MPs to move without huge elastic restraints due to the possible rearrangements of physical cross-links formed by PS chains. In this soft state of the polymer matrix, the magnetic field was turned on (point 3), which led to a sharp increase in the storage modulus and a decrease in the damping factor due to the magnetic interaction of the MPs and the formation of chain-like structures. This effect was much more pronounced than the magnetorheological effect at room temperature (Figure 2a,c). The temperature was then lowered (point 4) while the magnetic field was maintained to rearrange and reinforce the PS aggregates and “freeze” the MPs in their new positions. When the magnetic field was turned off (point 5), the storage modulus was almost twice the initial value, which is a typical difference between isotropic and anisotropic samples [23,24]. The formation of chain-like structures by MPs was observed using scanning electron microscopy (SEM). Figure 3d,e show the cross-section of the initial sample with random MP distribution and the sample with aligned MP distribution after magnetic field and temperature treatment. It is worth noting that the MP chains are quite short because the material does not reach its melting point, and better alignment can be achieved by using higher temperatures.

### 3.3. Magnetic Responses of Isotropic and Anisotropic Samples

**Figure 4:**
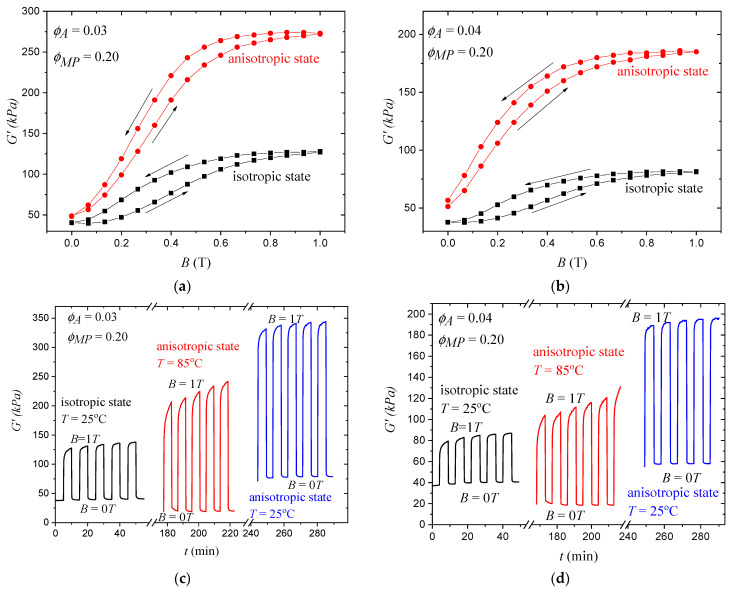
Magnetic response of initial isotropic and anisotropic samples based on polymer matrices containing (**a**,**c**) 3% PS chains and (**b**,**d**) 4% PS chains: (**a**,**b**) show the field dependence of the storage modulus on increasing and decreasing magnetic field for the initial sample with isotropic distribution of MPs (black squares) and anisotropic sample with aligned MPs (red circles). The direction of the magnetic field change is indicated by arrows. The corresponding dependencies for other samples are presented in SI, Figures S6 and S7; (**c**,**d**) show the time dependence of MATEs with different MP distributions at different temperatures when the magnetic field is periodically turned on and off.

Not only did the storage modulus of the MATEs themselves change due to the formation of chain-like structures by the MPs, but also the magnetic response, which can be characterized by the relative increase in the storage modulus of the MATEs in the magnetic field. Figure 4a,b show the dependence of the storage modulus on the increase and decrease in the magnetic field for the initial sample with a uniform distribution of MPs and the oriented sample with aligned MPs. It is clearly seen that although the value of the off-field modulus of the oriented sample is higher, both its relative and absolute increase are greater for the oriented sample. This can be attributed to the stronger interaction of columnar-ordered MPs with each other in the magnetic field due to the smaller distance between the particles [69,70,71] and the change in the particle microstructure [72]. The hysteresis loop can be explained by the formation of a strong network of MPs in the magnetic field, which was not completely reformed when the magnetic field was turned off. Therefore, hysteresis was more pronounced in isotropic MATEs, because oriented ones came closer to this optimal network, which was formed to some extent. Figure 4c,d show the response of MATEs with different MP distributions at different temperatures to periodically turning on and off the magnetic field. The oriented sample exhibited the highest off-field and on-field storage moduli due to the reasons discussed earlier. While the MATE at a high temperature of 85 °C had the lowest off-field modulus due to the softer polymer matrix, it showed a higher on-field modulus than the initial sample due to fewer restrictions on the movement of MPs within the polymer medium. At room temperature, the samples were quite stable to periodic magnetic field cycles: They showed only a small change in their moduli with each magnetic field cycle, and the typical relaxation times to reach the plateau seemed to be a few minutes. At high temperatures, however, the time dependence of the MATE properties was more pronounced due to the greater freedom of the MPs inside the heated polymer matrix.

### 3.4. Programming of the MATEs’ Properties

**Figure 5:**
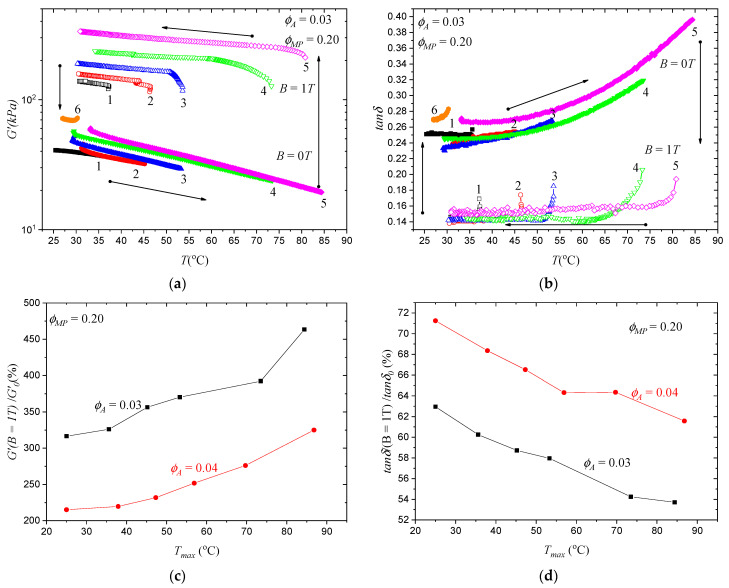
Programming of MATEs’ properties by using the magnetic field and different treatment temperatures. Temperature dependency of (**a**) storage modulus and (**b**) damping factor of MATEs with 3% PS and 20% MPs in heating cycles with no field and cooling in the magnetic field and an increase in the maximum temperature in each cycle. Arrows show the direction of temperature change and digits near the curves indicate the number of cycles. Similar curves for the samples with low MP concentrations are available in SI, Figures S8 and S9. Magnetic response considering the (**c**) storage modulus and (**d**) damping factor of MATEs with 20 vol% of MPs depending on treatment temperature. T = 25 °C refers to the initial sample. Different colors are for matrices with different volume fractions of the PS block as indicated.

We have already shown that it is possible to generate oriented MP structures inside MATEs at high temperatures and drastically change their properties and magnetic response. Due to the temperature-sensitive nature of the polymer matrix, it is also possible to fine-tune these properties by using different treatment temperatures. Figure 5a,b show the temperature dependence of the storage modulus and damping factor of the MATEs in MP orientation cycles similar to Figure 3 discussed above. First, the initial sample was heated to temperature T_max_ (solid symbols), and then the magnetic field B = 1T was turned on, and the sample was cooled to room temperature (empty symbols). Then, the magnetic field was turned off, and the cycle was repeated, with T_max_ increasing each time. It can be seen that the mechanical properties of the MATEs gradually improved with each cycle, both in the magnetic field and in the zero field, because each temperature increment softened the polymer matrix even more so that the MPs had fewer and fewer constraints to form an optimal structure. This allowed us to vary the properties of the sample over a wide range. Figure 5c,d show the dependence of the storage modulus and the damping factor relative change in the magnetic field on treatment temperature T_max_, where the first point of each curve corresponds to the initial state of the sample. Similar to the storage modulus, the magnetic response can also be tuned with treatment temperature due to the better reorganization of MPs at higher temperatures.

### 3.5. Reorientation of MP Structures and Reprogramming the MATEs’ Properties

**Figure 6:**
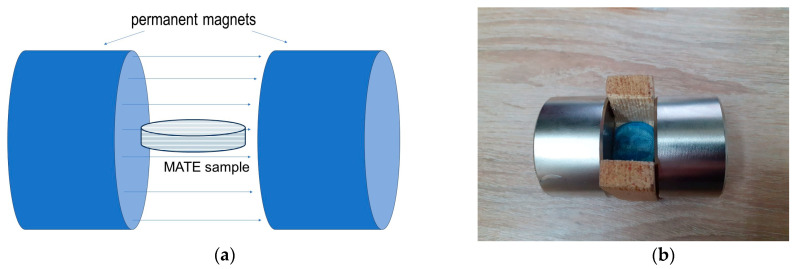
(**a**) Configuration for producing MATEs with magnetic structures oriented parallel to the surface of disk-like samples; (**b**) photo of a MATE sample confined within a blue plastic mold and placed between two permanent magnets to orient the MP structures parallel to the disc surface.

To demonstrate the possibility of reorienting the magnetic aggregates, MATE samples were subjected to additional heating cycles, followed by cooling in a magnetic field oriented parallel to the disc-shaped sample surface. The geometry of the rheometer did not allow for such reorientation, so instead we heated the sample in an oven with permanent NdFeB magnets attached to apply a magnetic field parallel to the surface of the disk-like samples (Figure 6). The heating temperature in this case was set to 85 °C, similar to the experiments discussed above. The sample was exposed to a parallel magnetic field during 2 h of heating and 30 min of cooling down to room temperature. After that, the permanent magnets were removed. The SEM image of the magnetic structures oriented parallel to the disk surface is shown in Supplementary Information, Figure S10. It should be noted that the magnetic field of the permanent magnets near the surface is only B ≈ 0.4T (measured with a magnetometer in the absence of the sample), which is considerably weaker than B = 1T used in our previous experiments. Moreover, the magnetic field generated by a pair of permanent magnets was not perfectly uniform. Hence, this reorientation method did not exactly replicate the reorientation in the rheometer.

**Figure 7:**
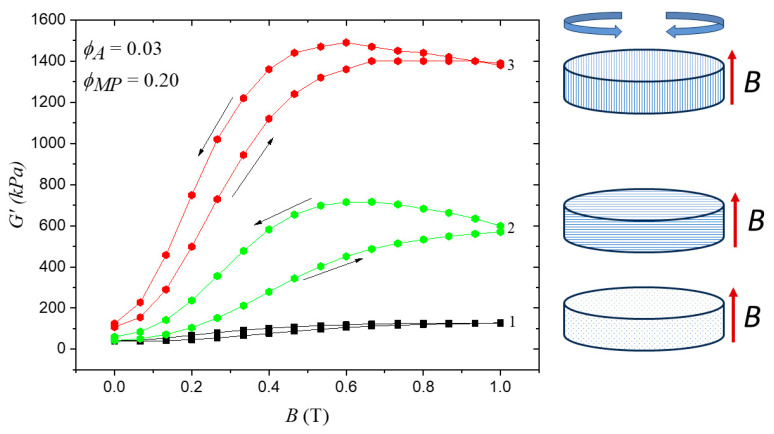
Magnetic response of MATE samples based on the polymer matrix with 3% of PS containing 20 vol% of MPs with different arrangements of MPs: 1 (black squares)–initial isotropic sample, 2 (green circles)–sample with parallel MP orientation, and 3 (red circles)–sample with perpendicular MP orientation. The figures on the right schematically show the MPs distributions inside disk-shaped samples and the orientation of the applied magnetic field B (red arrows).

We then measured the magnetic response of the sample treated this way. It should be stressed that the uniform magnetic field was applied perpendicular to the internal aggregate orientation. Unexpectedly, the increase in the storage modulus of the sample was significantly higher than that of the initial isotropic sample and the sample after the first temperature-field treatment performed in the rheometer (compare Figure 7 and Figure 4a). After orienting the magnetic structures parallel to the disk surface, the value of G′ increased almost twofold at the highest field of 1T. Therefore, we repeated the temperature and magnetic field treatment of the same sample again in the rheometer according to the protocol shown in Figure 3a and obtained the material with a novel structure of magnetic particles demonstrating a tremendous magnetic response. Figure 7 compares the room-temperature magnetic responses of the initial isotropic sample (curve 1) and the anisotropic samples with magnetic aggregates oriented parallel (curve 2) and perpendicular (curve 3) to the disk surface. One can see that the highest magnetic response is realized when the external magnetic field is oriented along the internal structure of the magnetic filler. This result is consistent with the data reported in [22,53,54,55,73].

It should be emphasized that the process of magnetic particle rearrangement at elevated temperatures is partially hindered by the fact that the A-g-B matrix is in an elastomeric state at the maximum treatment temperature of 100 °C. In addition, the resulting magnetic particle structures depend on their pretreatment orientation. In particular, the average distances between magnetic particles in isotropic composites can be expected to be larger than the interparticle distance in chain-like structures formed by heating and cooling the sample in a magnetic field. The smaller the distance, the stronger the interaction between the particles in the magnetic field, and thus the more effective their restructuring. Magnetic particles within the preoriented samples exposed to a perpendicular magnetic field respond as aggregates, in contrast to isolated particles. Moreover, the restructuring of the magnetic filler is highly time-dependent due to the relaxation of the A-g-B matrix, which is locally stressed by particle displacements from their initial positions in the applied magnetic field. This may explain the significant increase in the magnetic response of the material with a prealigned arrangement of magnetic particles.

## 4. Discussion

The results obtained demonstrate that the A-g-B brush platform provides a promising route to produce magnetoactive thermoplastic elastomers with programmable and reprogrammable viscoelasticity and magnetic response. In contrast to chemically cross-linked polymer networks, the A-g-B brushes comprise active dispersing media that not only exhibit thermosensitive viscoelastic properties but also provide the possibility to restructure magnetic particles. The latter can be achieved by applying magnetic fields of different orientations at elevated temperatures, even below the melting temperature of the A-g-B matrix. Cooling to room temperature results in a material with oriented magnetic aggregates trapped in a physical network. It was found that the temperature field treatment led to an improvement in the magnetic response of MATEs by two orders of magnitude when the magnetic field and magnetic aggregates were colinear.

The results of this work demonstrate that thermoplastic elastomers are promising for the in situ restructuring of magnetic particles. The A-g-B-based MATEs have multiple advantages compared to conventional thermoplastic elastomers: (i) At room temperature, the brush structure provides tissue-mimetic softness and strain stiffening; (ii) upon heating, the dissociation of A-g-B networks occurs at moderate temperatures controlled by copolymer architecture; and (iii) in a melt state, the compact conformations of disentangled brush molecules lead to much lower viscosity than their linear counterparts [74].

An ability to reprogram material properties in situ impacts the future of soft robotics and medical devices. In addition, the thermoplastic nature of the A-g-B matrix enables injection molding and the 3D printing of the magnetic composites, preventing particle sedimentation [30].

## Data Availability

The data presented in this study are available upon request from the corresponding authors.

## Supplementary Materials

The following supporting information can be downloaded at: https://www.mdpi.com/article/10.3390/polym15234607/s1, Figure S1: 1H NMR methacrylate functionalized polystyrene macromonomer; Figure S2: 1H NMR of PDMS-nBA-PS A-g-B network title; Figure S3: Amplitude dependence of the storage modulus and damping factor of MATEs with different MP concentrations. Figure S4: Temperature dependence of the storage modulus and the damping factor for MATEs based on polymer matrix containing 3% PS chains; Figure S5: Temperature dependence of the storage modulus and the damping factor for MATEs based on polymer matrix containing 4% PS chains; Figure S6: Dependence of the storage modulus and damping factor on the increase and decrease in magnetic field for the initial sample with an isotropic distribution of MPs and the oriented sample with aligned MPs based on the matrix with 3% PS; Figure S7: Dependence of the storage modulus and damping factor on the increase and decrease in the magnetic field for the initial sample with isotropic distribution of MPs and the oriented sample with aligned MPs based on the matrix with 4% PS; Figures S8 and S9: Programming of the MATEs’ properties; Figure S10: SEM image of the sample with 20% MPs based on polymer matrix with 3% fraction of PS-block after treatment in magnetic field parallel to the sample surface.

## References

[1] Ubaidillah, Sutrisno J., Purwanto A., Mazlan S.A. (2015). Recent Progress on Magnetorheological Solids: Materials, Fabrication, Testing, and Applications. Adv. Eng. Mater..

[2] Choi S.B., Li W., Yu M., Du H., Fu J., Do P.X. (2016). State of the Art of Control Schemes for Smart Systems Featuring Magneto-Rheological Materials. Smart Mater. Struct..

[3] Filipcsei G., Csetneki I., Szilágyi A., Zrínyi M., Gong B., Sanford A.R., Ferguson J.S. (2007). Magnetic Field-Responsive Smart Polymer Composites. Oligomers-Polymer Composites-Molecular Imprinting.

[4] Shamonin M., Kramarenko E.Y., Domracheva N., Caporali M., Rentschler E. (2018). Chapter 7—Highly Responsive Magnetoactive Elastomers. Novel Magnetic Nanostructures.

[5] Nguyen V.Q., Ahmed A.S., Ramanujan R.V. (2012). Morphing Soft Magnetic Composites. Adv. Mater..

[6] Bastola A.K., Paudel M., Li L., Li W. (2020). Recent Progress of Magnetorheological Elastomers: A Review. Smart Mater. Struct..

[7] Cantera M.A., Behrooz M., Gibson R.F., Gordaninejad F. (2017). Modeling of Magneto-Mechanical Response of Magnetorheo-logical Elastomers (MRE) and MRE-Based Systems: A Review. Smart Mater. Struct..

[8] Lopez-Lopez M.T., Durán J.D.G., Iskakova L.Y., Zubarev A.Y. (2016). Mechanics of Magnetopolymer Composites: A Review. J. Nanofluids.

[9] Odenbach S. (2016). Microstructure and Rheology of Magnetic Hybrid Materials. Arch. Appl. Mech..

[10] Menzel A.M. (2015). Tuned, Driven, and Active Soft Matter. Phys. Rep..

[11] Galipeau E., Ponte Castañeda P. (2013). Giant Field-Induced Strains in Magnetoactive Elastomer Composites. Proc. R. Soc. A Math. Phys. Eng. Sci..

[12] Saveliev D.V., Belyaeva I.A., Chashin D.V., Fetisov L.Y., Romeis D., Kettl W., Kramarenko E.Y., Saphiannikova M., Stepanov G.V., Shamonin M. (2020). Giant Extensional Strain of Magnetoactive Elastomeric Cylinders in Uniform Magnetic Fields. Materials.

[13] Glavan G., Belyaeva I.A., Drevenšek-Olenik I., Shamonin M. (2023). Experimental Study of Longitudinal, Transverse and Volume Strains of Magnetoactive Elastomeric Cylinders in Uniform Magnetic Fields. J. Magn. Magn. Mater..

[14] Sánchez P.A., Stolbov O.V., Kantorovich S.S., Raikher Y.L. (2019). Modeling the Magnetostriction Effect in Elastomers with Magnetically Soft and Hard Particles. Soft Matter.

[15] Ginder J.M., Clark S.M., Schlotter W.F., Nichols M.E. (2002). Magnetostrictive Phenomena in Magnetorheological Elastomers. Int. J. Mod. Phys. B.

[16] Bednarek S. (1999). The Giant Magnetostriction in Ferromagnetic Composites within an Elastomer Matrix. Appl. Phys. A.

[17] Kostrov S.A., Shamonin M., Stepanov G.V., Kramarenko E.Y. (2019). Magnetodielectric Response of Soft Magnetoactive Elastomers: Effects of Filler Concentration and Measurement Frequency. Int. J. Mol. Sci..

[18] Moucka R., Sedlacik M., Cvek M. (2018). Dielectric Properties of Magnetorheological Elastomers with Different Microstructure. Appl. Phys. Lett..

[19] Bica I., Liu Y.D., Choi H.J. (2012). Magnetic Field Intensity Effect on Plane Electric Capacitor Characteristics and Viscoelasticity of Magnetorheological Elastomer. Colloid Polym. Sci..

[20] Stepanov G.V., Semerenko D.A., Bakhtiiarov A.V., Storozhenko P.A. (2013). Magnetoresistive Effect in Magnetoactive Elastomers. J. Supercond. Nov. Magn..

[21] Winger J., Schümann M., Kupka A., Odenbach S. (2019). Influence of the Particle Size on the Magnetorheological Effect of Magnetorheological Elastomers. J. Magn. Magn. Mater..

[22] Nam T.H., Petríková I., Marvalová B. (2020). Experimental Characterization and Viscoelastic Modeling of Isotropic and Anisotropic Magnetorheological Elastomers. Polym. Test..

[23] Sorokin V.V., Ecker E., Stepanov G.V., Shamonin M., Monkman G.J., Kramarenko E.Y., Khokhlov A.R. (2014). Experimental Study of the Magnetic Field Enhanced Payne Effect in Magnetorheological Elastomers. Soft Matter.

[24] Kostrov S.A., Gorodov V.V., Muzafarov A.M., Kramarenko E.Y. (2022). Comparative Analysis of Magnetorheological Effect in Soft Isotropic and Anisotropic Magnetoactive Elastomers. Polym. Sci. Ser. B.

[25] Shen Y., Golnaraghi M.F., Heppler G.R. (2004). Experimental Research and Modeling of Magnetorheological Elastomers. J. Intell. Mater. Syst. Struct..

[26] Bellan C., Bossis G. (2002). Field Dependence of Viscoelastic Properties of MR Elastomers. Int. J. Mod. Phys. B.

[27] Lokander M., Stenberg B. (2003). Performance of Isotropic Magnetorheological Rubber Materials. Polym. Test..

[28] Li W.H., Zhou Y., Tian T.F. (2010). Viscoelastic Properties of MR Elastomers under Harmonic Loading. Rheol. Acta.

[29] Chiba N., Yamamoto K., Hojo T., Kawai M., Mitsumata T. (2013). Wide-Range Modulation of Dynamic Modulus and Loss Tangent for Magnetic Elastomers Containing Submilimeter Magnetic Particles. Chem. Lett..

[30] Bira N., Dhagat P., Davidson J.R. (2020). A Review of Magnetic Elastomers and Their Role in Soft Robotics. Front. Robot. AI.

[31] Hu W., Lum G.Z., Mastrangeli M., Sitti M. (2018). Small-Scale Soft-Bodied Robot with Multimodal Locomotion. Nature.

[32] Makarova L.A., Alekhina Y.A., Rusakova T.S., Perov N.S. (2016). Tunable Properties of Magnetoactive Elastomers for Biomedical Applications. Phys. Procedia.

[33] Hooshiar A., Payami A., Dargahi J., Najarian S. (2021). Magnetostriction-Based Force Feedback for Robot-Assisted Cardiovascular Surgery Using Smart Magnetorheological Elastomers. Mech. Syst. Signal Process..

[34] Makarova L.A., Alekhina Y.A., Isaev D.A., Khairullin M.F., Perov N.S. (2021). Tunable Layered Composites Based on Magnetoactive Elastomers and Piezopolymer for Sensors and Energy Harvesting Devices. J. Phys. D Appl. Phys..

[35] Xu T., Zhang J., Salehizadeh M., Onaizah O., Diller E. (2019). Millimeter-Scale Flexible Robots with Programmable Three-Dimensional Magnetization and Motions. Sci. Robot..

[36] Shinoda H., Azukizawa S., Maeda K., Tsumori F. (2019). Bio-Mimic Motion of 3D-Printed Gel Structures Dispersed with Magnetic Particles. J. Electrochem. Soc..

[37] Ren Z., Hu W., Dong X., Sitti M. (2019). Multi-Functional Soft-Bodied Jellyfish-like Swimming. Nat. Commun..

[38] Liao Z., Zoumhani O., Boutry C.M. (2023). Recent Advances in Magnetic Polymer Composites for BioMEMS: A Review. Materials.

[39] Li Y., Li J., Li W., Du H. (2014). A State-of-the-Art Review on Magnetorheological Elastomer Devices. Smart Mater. Struct..

[40] Stoll A., Mayer M., Monkman G.J., Shamonin M. (2014). Evaluation of Highly Compliant Magneto-Active Elastomers with Colossal Magnetorheological Response. J. Appl. Polym. Sci..

[41] Grosberg A.Y., Khokhlov A.R. (1989). Statistical Physics of Macromolecules.

[42] Stepanov G.V., Borin D.Y., Kramarenko E.Y., Bogdanov V.V., Semerenko D.A., Storozhenko P.A. (2014). Magnetoactive Elastomer Based on Magnetically Hard Filler: Synthesis and Study of Viscoelastic and Damping Properties. Polym. Sci. Ser. A.

[43] Kostrov S.A., Dashtimoghadam E., Keith A.N., Sheiko S.S., Kramarenko E.Y. (2021). Regulating Tissue-Mimetic Mechanical Properties of Bottlebrush Elastomers by Magnetic Field. ACS Appl. Mater. Interfaces.

[44] Tian T., Nakano M. (2018). Fabrication and Characterisation of Anisotropic Magnetorheological Elastomer with 45° Iron Particle Alignment at Various Silicone Oil Concentrations. J. Intell. Mater. Syst. Struct..

[45] Li W.H., Zhang X.Z. (2010). A Study of the Magnetorheological Effect of Bimodal Particle Based Magnetorheological Elastomers. Smart Mater. Struct..

[46] Schmauch M.M., Mishra S.R., Evans B.A., Velev O.D., Tracy J.B. (2017). Chained Iron Microparticles for Directionally Controlled Actuation of Soft Robots. ACS Appl. Mater. Interfaces.

[47] Ding L., Zhang J., Shu Q., Liu S., Xuan S., Gong X., Zhang D. (2021). Magnetism-Responsive Anisotropic Film with Self-Sensing and Multifunctional Shape Manipulation. ACS Appl. Mater. Interfaces.

[48] Lin D., Yang F., Gong D., Lin Z., Li R., Qian W., Li C., Jia S., Chen H. (2021). Magnetoactive Soft Drivers with Radial-Chain Iron Microparticles. ACS Appl. Mater. Interfaces.

[49] Lu H., Zhang M., Yang Y., Huang Q., Fukuda T., Wang Z., Shen Y. (2018). A Bioinspired Multilegged Soft Millirobot That Functions in Both Dry and Wet Conditions. Nat. Commun..

[50] Farshad M., Benine A. (2004). Magnetoactive Elastomer Composites. Polym. Test..

[51] Puente-Córdova J.G., Reyes-Melo M.E., Palacios-Pineda L.M., Martínez-Perales I.A., Martínez-Romero O., Elías-Zúñiga A. (2018). Fabrication and Characterization of Isotropic and Anisotropic Magnetorheological Elastomers, Based on Silicone Rubber and Carbonyl Iron Microparticles. Polymers.

[52] Sohoni G.B., Mark J.E. (1987). Anisotropic Reinforcement in Elastomers Containing Magnetic Filler Particles. J. Appl. Polym. Sci..

[53] Sun T.L., Gong X.L., Jiang W.Q., Li J.F., Xu Z.B., Li W.H. (2008). Study on the Damping Properties of Magnetorheological Elastomers Based on Cis-Polybutadiene Rubber. Polym. Test..

[54] Wu J., Gong X., Fan Y., Xia H. (2010). Anisotropic Polyurethane Magnetorheological Elastomer Prepared through in Situ Polycondensation under a Magnetic Field. Smart Mater. Struct..

[55] Kaleta J., Królewicz M., Lewandowski D. (2011). Magnetomechanical Properties of Anisotropic and Isotropic Magnetorheological Composites with Thermoplastic Elastomer Matrices. Smart Mater. Struct..

[56] Varga Z., Filipcsei G., Zrínyi M. (2005). Smart Composites with Controlled Anisotropy. Polymer.

[57] Hajsz T., Csetneki I., Filipcsei G., Zrinyi M. (2006). Swelling Kinetics of Anisotropic Filler Loaded PDMS Networks. Phys. Chem. Chem. Phys..

[58] Varga Z., Filipcsei G., Szilágyi A., Zrínyi M. (2005). Electric and Magnetic Field-Structured Smart Composites. Macromol. Symp..

[59] Ouchi S., Mitsumata T. (2009). Magnetorheological Effect of Magnetic Gels Containing Fe_2_O_3_. Trans. Mater. Res. Soc. Jpn..

[60] Boczkowska A., Awietjan S.F., Wroblewski R. (2007). Microstructure-Property Relationships of Urethane Magnetorheological Elastomers. Smart Mater. Struct..

[61] Berasategi J., Salazar D., Gomez A., Gutierrez J., Sebastián M.S., Bou-Ali M., Barandiaran J.M. (2020). Anisotropic Behaviour Analysis of Silicone/Carbonyl Iron Particles Magnetorheological Elastomers. Rheol. Acta.

[62] Chokkalingam R., Pandi R.S., Mahendran M. (2011). Magnetomechanical Behavior of Fe/PU Magnetorheological Elastomers. J. Compos. Mater..

[63] Coquelle E., Bossis G., Szabo D., Giulieri F. (2006). Micromechanical Analysis of an Elastomer Filled with Particles Organized in Chain-like Structure. J. Mater. Sci..

[64] Mitsumata T., Nagata A., Sakai K., Taniguchi T. (2004). Anisotropy in Storage Modulus of Magnetic Gels Induced by Magnetization. Jpn. J. Appl. Phys..

[65] Komarov P.V., Khalatur P.G., Khokhlov A.R. (2021). Magnetoresponsive Smart Nanocomposites with Highly Cross-Linked Polymer Matrix. Polym. Adv. Technol..

[66] Romeis D., Toshchevikov V., Saphiannikova M. (2016). Elongated Micro-Structures in Magneto-Sensitive Elastomers: A Dipolar Mean Field Model. Soft Matter.

[67] Kostrov S.A., Maw M.R., Sheiko S.S., Kramarenko E.Y. (2023). Magnetoactive Thermoplastic Elastomers with Bottlebrush Strands: Switching and Programming of Mechanical Properties by a Magnetic Field. ACS Appl. Polym. Mater..

[68] Dashtimoghadam E., Maw M., Keith A.N., Vashahi F., Kempkes V., Gordievskaya Y.D., Kramarenko E.Y., Bersenev E.A., Nikitina E.A., Ivanov D.A. (2022). Super-Soft, Firm, and Strong Elastomers toward Replication of Tissue Viscoelastic Response. Mater. Horiz..

[69] Chen L., Gong X.L., Li W.H. (2007). Microstructures and Viscoelastic Properties of Anisotropic Magnetorheological Elastomers. Smart Mater. Struct..

[70] Ivaneyko D., Toshchevikov V., Saphiannikova M. (2018). Dynamic-Mechanical Behaviour of Anisotropic Magneto-Sensitive Elastomers. Polymer.

[71] Asadi Khanouki M., Sedaghati R., Hemmatian M. (2019). Experimental Characterization and Microscale Modeling of Isotropic and Anisotropic Magnetorheological Elastomers. Compos. Part B Eng..

[72] Chougale S., Romeis D., Saphiannikova M. (2022). Magneto-Mechanical Enhancement of Elastic Moduli in Magnetoactive Elastomers with Anisotropic Microstructures. Materials.

[73] Abramchuk S., Kramarenko E., Stepanov G., Nikitin L.V., Filipcsei G., Khokhlov A.R., Zrínyi M. (2007). Novel Highly Elastic Magnetic Materials for Dampers and Seals: Part I. Preparation and Characterization of the Elastic Materials. Polym. Adv. Technol..

[74] Dashtimoghadam E., Fahimipour F., Keith A.N., Vashahi F., Popryadukhin P., Vatankhah-Varnosfaderani M., Sheiko S.S. (2021). Injectable Non-Leaching Tissue-Mimetic Bottlebrush Elastomers as an Advanced Platform for Reconstructive Surgery. Nat. Commun..

