# Supplementary material for: Programming and Reprogramming the Viscoelasticity and Magnetic Response of Magnetoactive Thermoplastic Elastomers
Source: Polymers (Basel). 2023 Dec 3;15(23):4607. doi: 10.3390/polym15234607 (PMC10708547; doi:10.3390/polym15234607)
Supplement: Supplementary file 1 [file polymers-15-04607-s001.zip › polymers-2725826-supplementary.pdf]

polymers

MDPI

Supporting information to Article

Programming and reprogramming the viscoelasticity and magnetic response of magnetoactive thermoplastic elastomers

Sergei A. Kostrov [1], Josiah H. Marshall [2], Mitchell Maw [2], Sergei S. Sheiko [2,*] and Elena Yu. Kramarenko [1,*]

[1] Faculty of Physics, Lomonosov Moscow State University, Leninskie Gory 1/2, 119991 Moscow, Russia; kostrov@polly.phys.msu.ru
[2] Department of Chemistry, University of North Carolina at Chapel Hill, Chapel Hill, NC 27599, USA; jmarshall@unc.edu (J.H.M.); mitrmaw5@live.unc.edu (M.M.)
* Correspondence: sergei@email.unc.edu (S.S.S.); kram@polly.phys.msu.ru (E.Y.K.)

PS-60 macromonomer synthesis:

All commerically purchased monomers were passed through basica alumina to remoe inhibitors before use in reactions. Polystrene macromonomer was synthesized via atom transfter radical polymerization (ATRP) with a targeted DP of 60. 20.0 g (192.03 mmol) of uninhibited sytrene monomer, 886.6 mg (4.2 mmol) of 2-hydroxyethyl 2-bromoisobutyrate (HEBIB) initiator, 416.0 mg (2.4 mmol) of N, N,N′,N″,N″-Pentamethyldiethylenetriame (PMDETA) ligand, and 30 g toluene were added to a 100 mL schlenk flask equipped with stir bar. The contents were then purged with N_2 gas for 1 hour before quickly adding 344.34 mg (2.4 mmol) of Cu(I)Br catalyst to the flask. Upon addition of the catalyst, the flask was purged with N_2 for an additional 15 minutes before placing the sealed flask into a hot oil bath set at 110 °C. The reaction proceeded for 5 hours before being stopped at 78% conversion. Unreacted monomers were removed by crashing the resulting polymer out of solution with methanol. After decanting the liquid from the polymer mass, the process of just dissolving the polymer in THF and crashing in methanol was repeated 3 times. The PS-OH macromonomer was air dried until a fine powder was obtained. In a subsequent reaction, 10.08 g (1.51 mmol) of the PS-OH macro and 30 mL of anhydrous dichloromethane were added to an oven dried 100 mL round-bottom flask equipped with stir bar. 0.281 g (1.81 mmol) of 2-isocyanatoethyl methacrylate (IEM) was added dropwise to the PS-OH solution along with 0.19 g (0.297 mmol) of dibutyltin dilaurate (DIBTL) catalyst. The reaction was stirred at room temperature for 24 hours before reaching full conversion. To remove unreacted IEM from the system, the post ATRP polymerization wash procedure was repeated (4X total washes). Figure S1 shows a ^{1}H NMR of the polystyrene macromonomer after being methacrylated with IEM. All NMR spectra were acquired on a Bruker Avance NEO 600 MHz spectrometer.

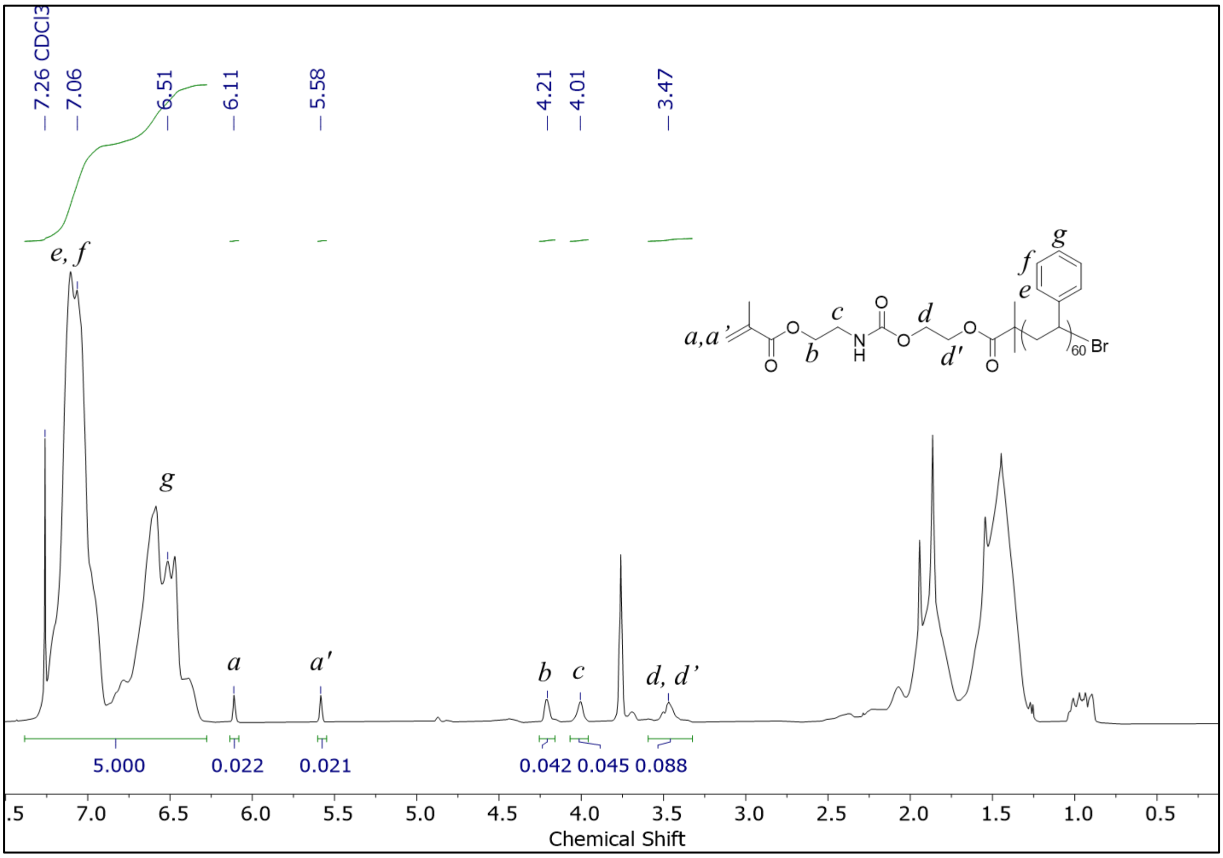

Figure S1. ^{1}H NMR methacrylate functionalized polystyrene macromonomer. (600 MHz, CDCl3): $\delta_{a,a'}$: 6.11 & 5.58 (sp^2-vinyl, 2H, s), $\delta_{b,c}$: 4.21 & 4.01((C=O)-O-C**H**$_2$-C**H**$_2$-NH-, 4H, d), $\delta_{d,d'}$: 3.47 (NH-(C=O)-O-C**H**$_2$-C**H**$_2$-O-, 4H, bs), $\delta_{d,e,f}$: 6.28-7.38 (polystyrene sp^2 aromatic, 5H, m).

PDMS-nBA-PS A-g-B synthesis:

A-g-B network was prepared via UV-initiated free radical polymerization. 12 g (13.3 mmol) of PDMS (Gelest® MCR-M11), 11.95 g (93.4 mmol) of n-BA, 0.741 g (0.112 mmol) - 3 % & 0.998 g (0.151 mmol) - 4 % of PS-macro, 60 g of toluene, and a stir bar were added to a 100 mL schlenk flask. The flask was covered in aluminum foil and 19 mg (0.045 mmol) Phenylbis(2,4,6-trimethylbenzoyl)phosphine oxide (BAPO) was added. Next, the flask was purged with N_2 for 1.5 hrs before removing the foil and exposing the contents to ambient light. The reaction was stirred at room temperature for 24 hrs before terminating the reaction with oxygen. Unreacted monomers were removed by crashing the resulting polymer out of solution with methanol. After decanting the liquid from the polymer mass, the process of just dissolving the polymer in toluene and crashing in methanol was repeated 3 times. Figure S2 shows ^{1}H NMR of the pure A-g-B system.

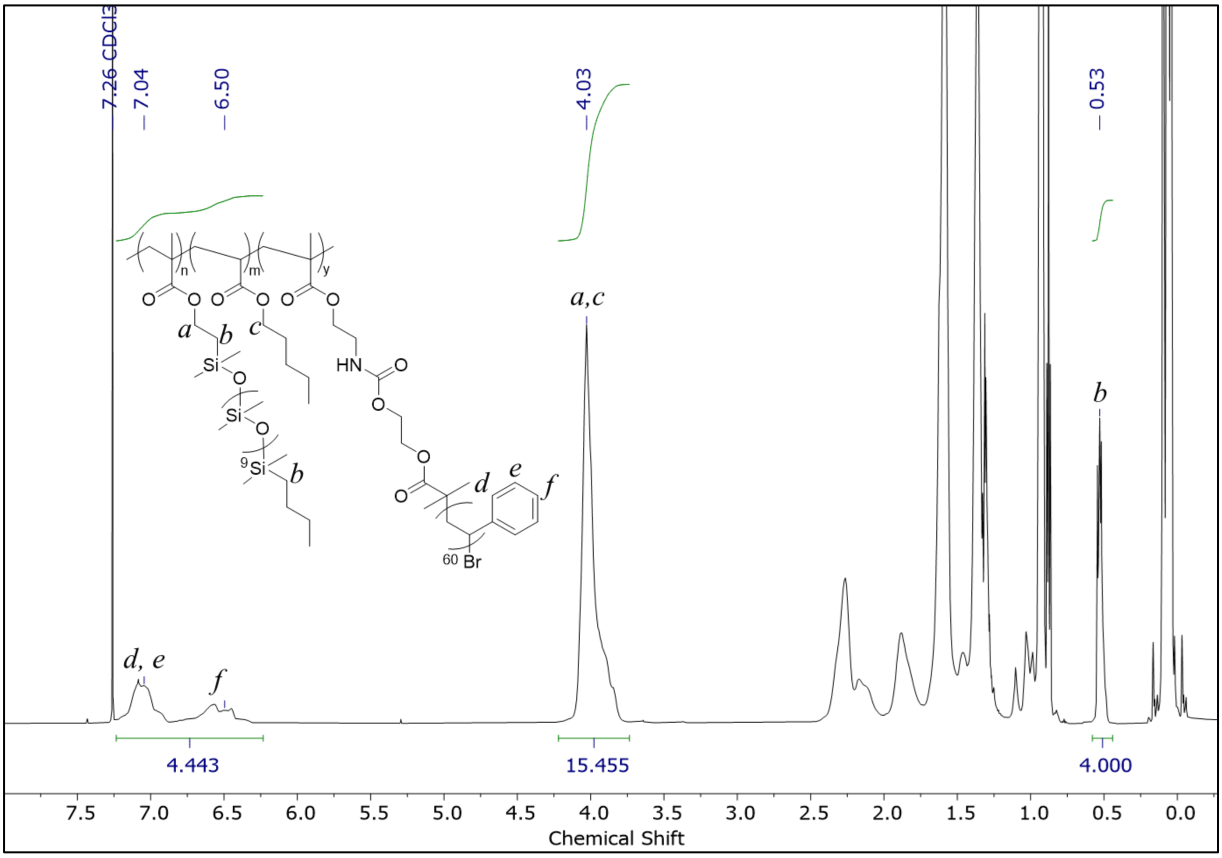

Figure S2. ^{1}H NMR of PDMS-nBA-PS A-g-B network. (600 MHz, $CDCl_3$): δ_a, δ_c: 4.03 ((C=O)-O-C**H**$_2$-, 4H, t), δ_b: 0.53 ((C=O)-O-C**H**$_2$- & Si-C**H**$_2$-, 4H, t), $\delta_{d,e,f}$: 6.32-7.31 ((polystyrene sp^2-aromatic, 5H, m). $n_g = 1 + \frac{((area\ a,c) - a)}{(area\ \ c)} = 1 + \frac{(15.455-2)}{2} = 7.7 \sim 8.$ $n_x = \frac{1}{\left[\frac{(Area\ d,e,f)}{(DP\ PS\ macro\ x\ 5)}\right]} = \frac{1}{\left[\frac{4.443}{60\ x\ 5}\right]} = \frac{1}{0.01481} = 67.5 \sim 68.$

Amplitude tests:

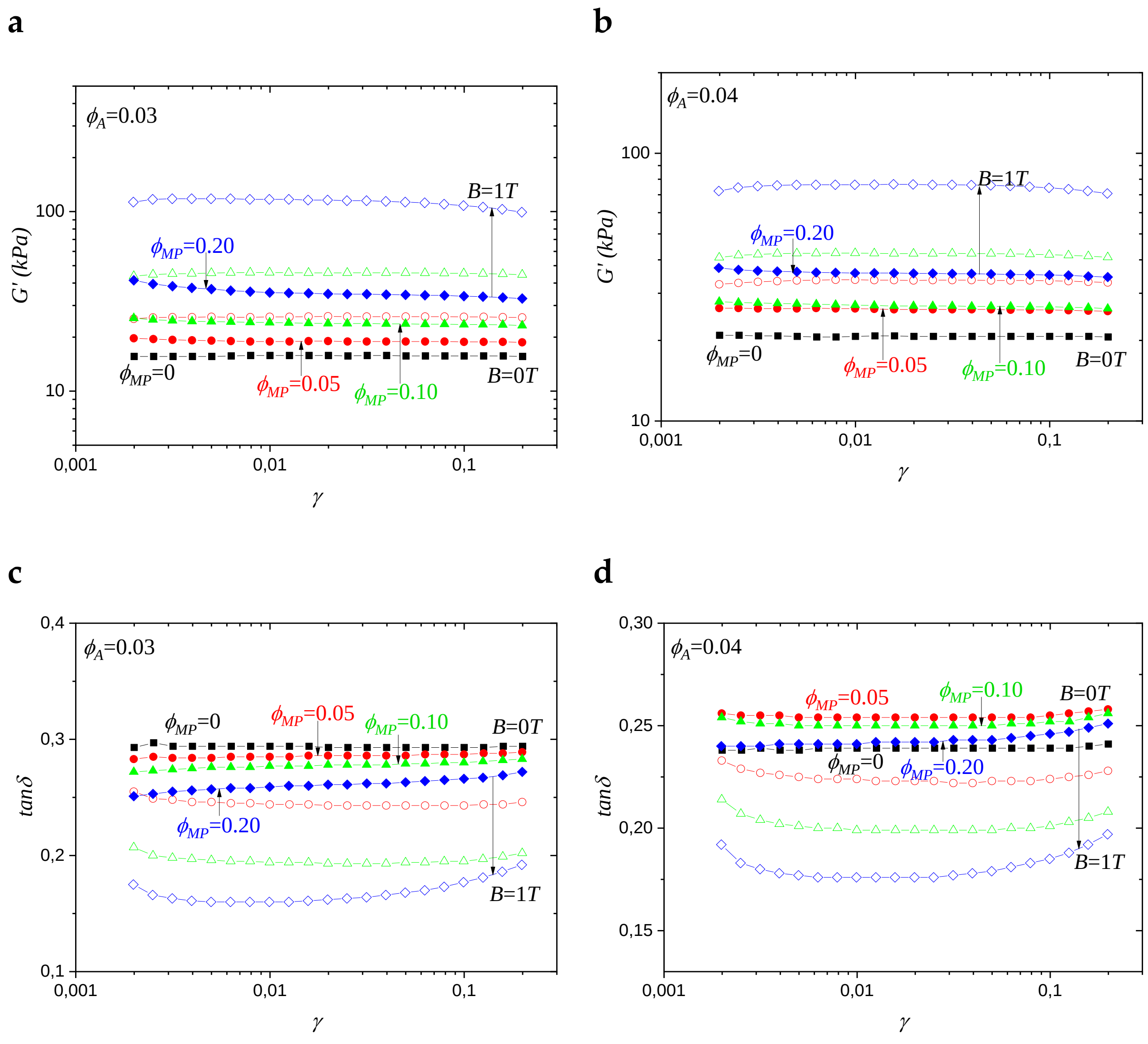

Figure S3. Amplitude dependence of (a,b) storage modulus and (c,d) damping factor of MATEs with different MPs concentration based on polymer with (a,c) 3% PS chains and (b,d) 4% PS chains.

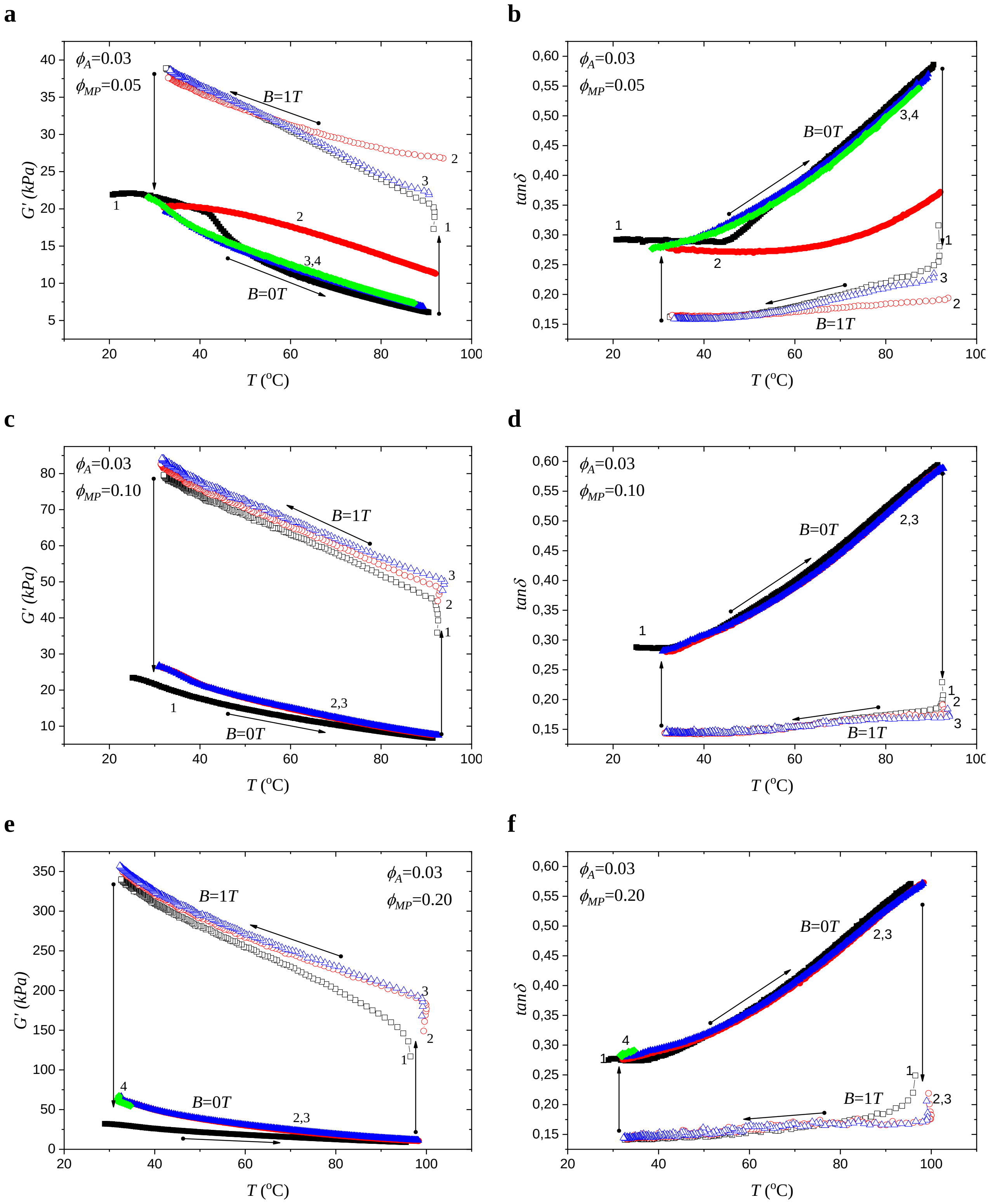

Figure S4. Temperature dependence of (a,c,e) storage modulus and (b,d,f) damping factor of MATEs with (a,b) 5% MPs, (c,d) 10% MPs and (e,f) 20% MPs based on polymer matrix containing 3% PS chains. Arrows show the direction of temperature change and digits near the curves indicate number of cycle. Experiments are similar to the one represented on fig.4(a,b) but include 3 cycles of MATE treatment. It is clearly seen from storage modulus dependence that the 1st heating curve differs from following ones because this curve correspond to initial state of the sample with isotropic distribution of MPs while next cycles correspond to anisotropic sample.

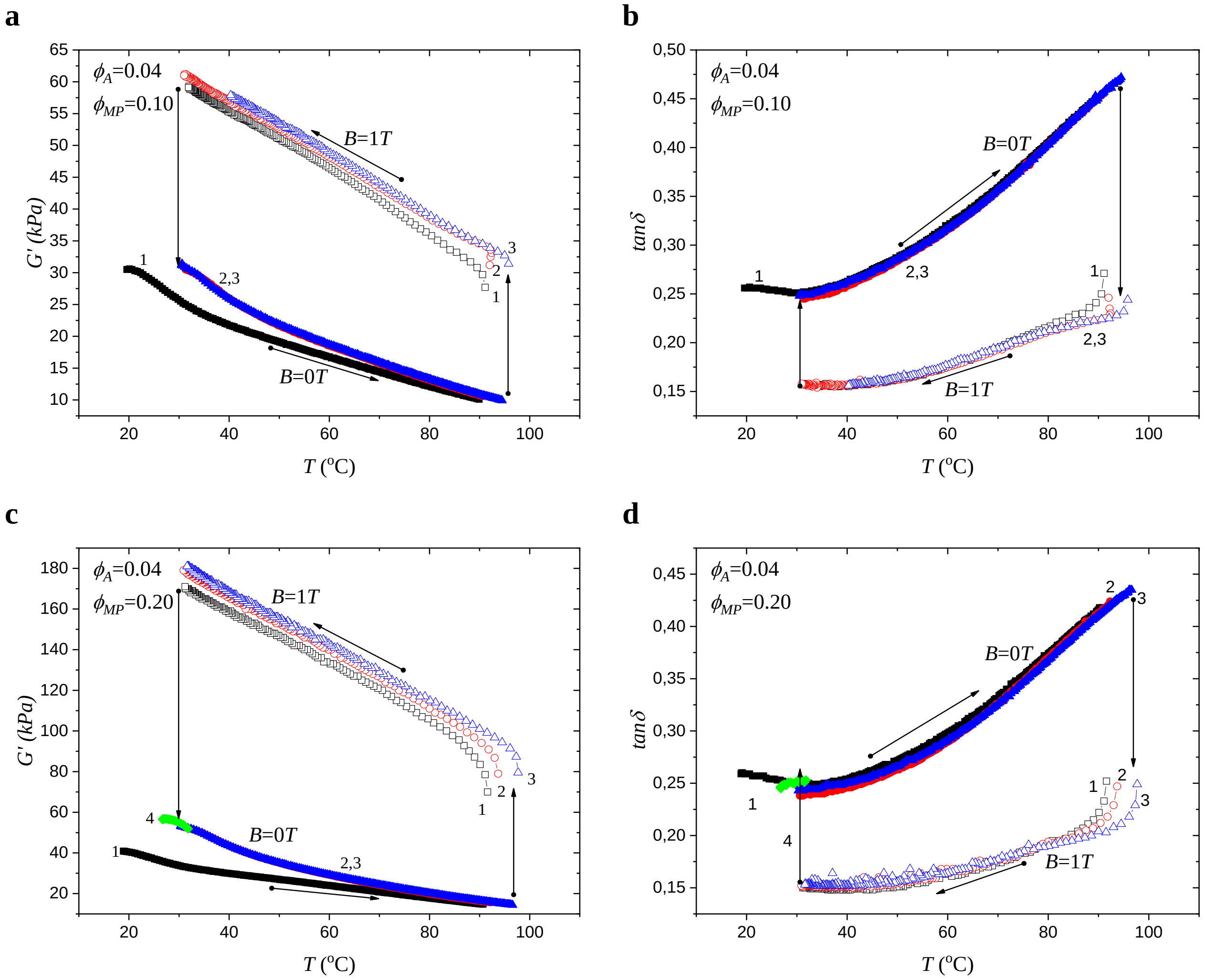

Figure S5. Temperature dependence of (a,c) storage modulus and (b,d) damping factor for MATEs with (a,b) 5% MPs and (c,d) 20% MPs based on polymer matrix containing 4% PS chains. Arrows show the direction of temperature change and digits near the curves indicate number of cycle. Experiments are similar to the one represented on fig.4(a,b) but include 3 cycles of of MATE treatment. It is clearly seen from storage modulus dependence that the 1st heating curve differs from following ones because this curve correspond to initial state of the sample with isotropic distribution of MPs while next cycles correspond to anisotropic sample.

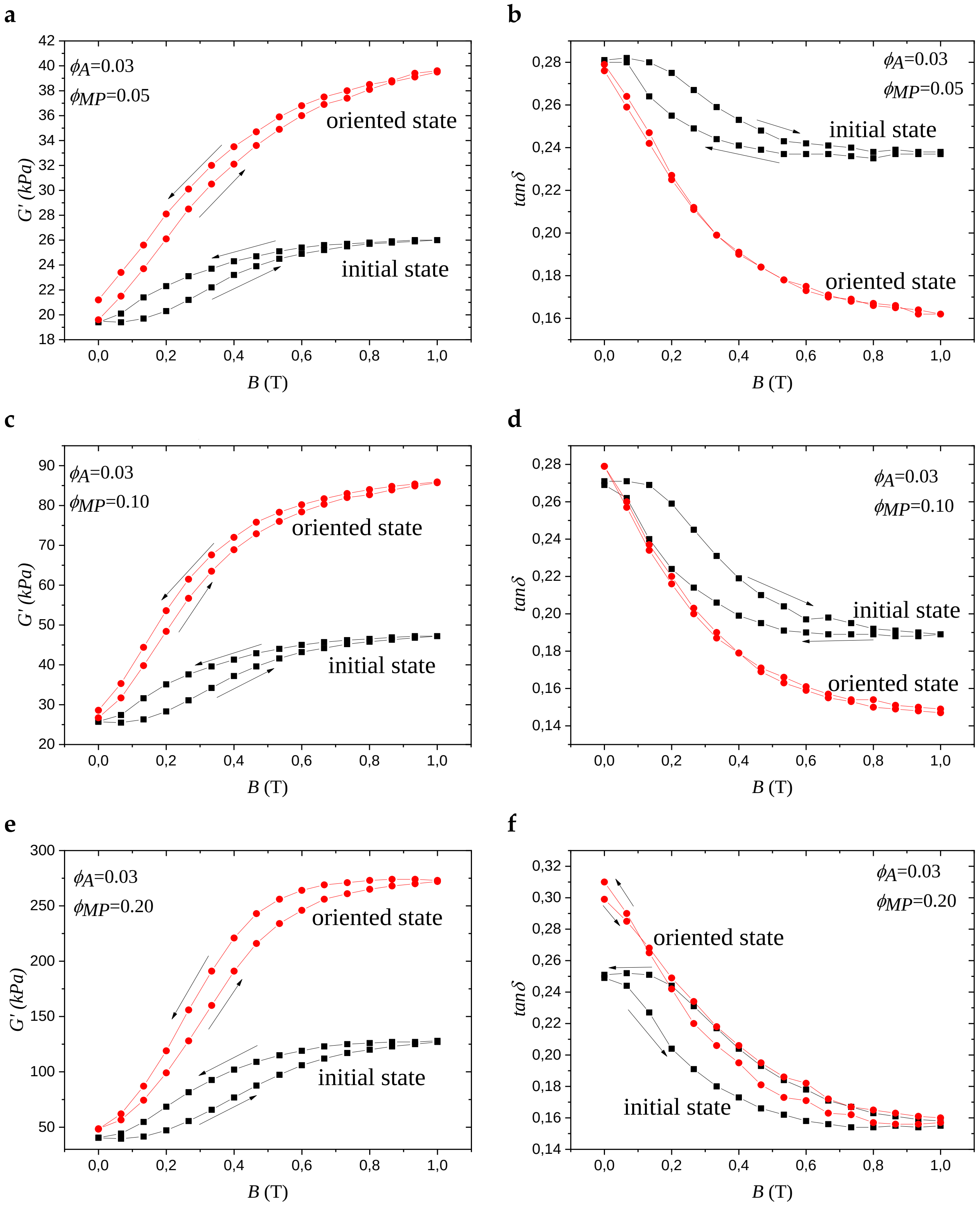

Figure S6. Dependence of (a,c,e) storage modulus and (b,d,f) damping factor on increasing and decreasing magnetic field for initial sample with even distribution of MPs (black line) and oriented sample with aligned MPs (red line). Direction of magnetic field change is shown with arrows. MATE is based on polymer with 3% PS chains

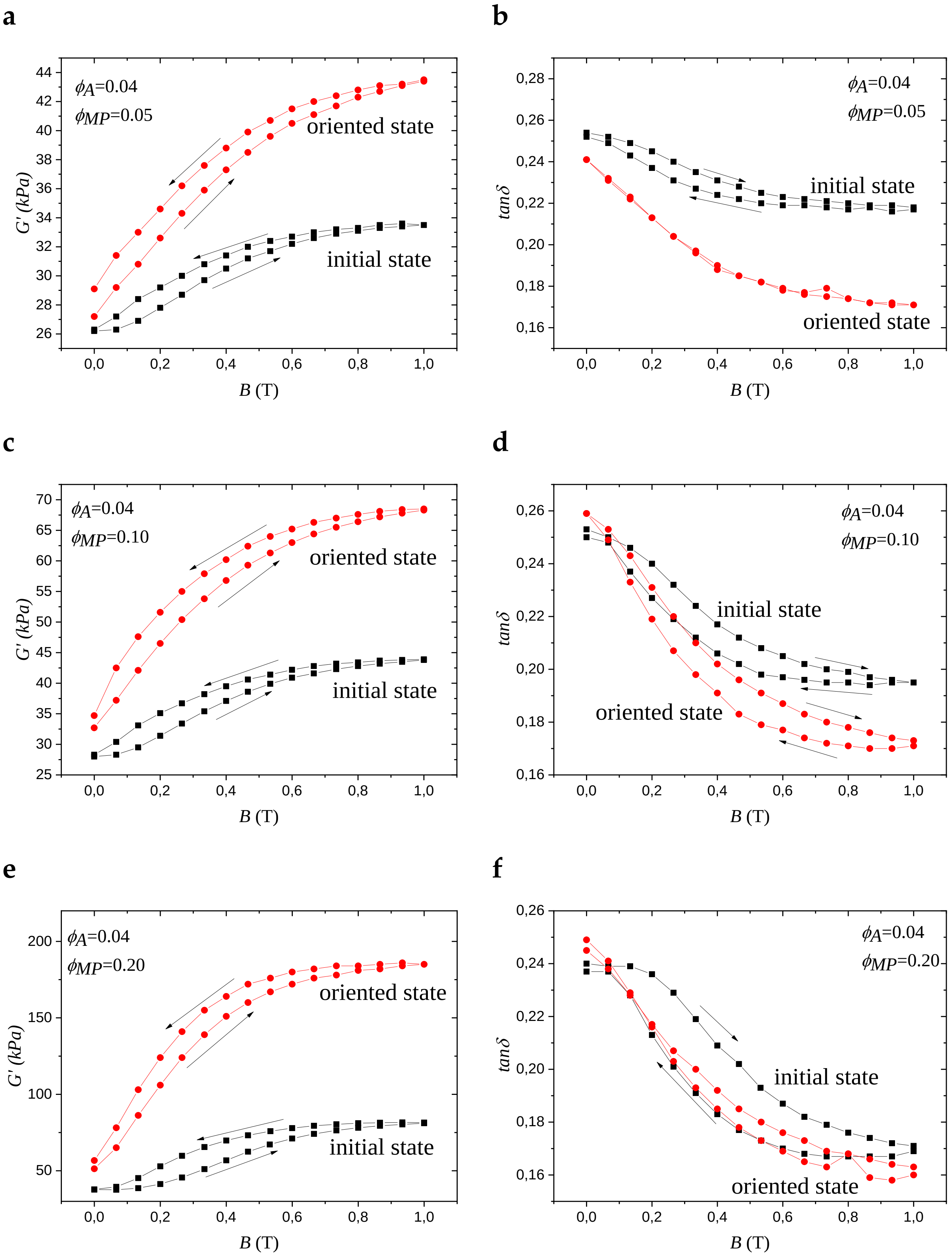

Figure S7. Dependency of (a,c,e) storage modulus and (b,d,f) damping factor on increasing and decreasing magnetic field for initial sample with even distribution of MPs (black line) and oriented sample with aligned MPs (red line). Direction of magnetic field change is shown with arrows. MATE is based on polymer with 4% PS chains.

Programming of the MATEs' properties:

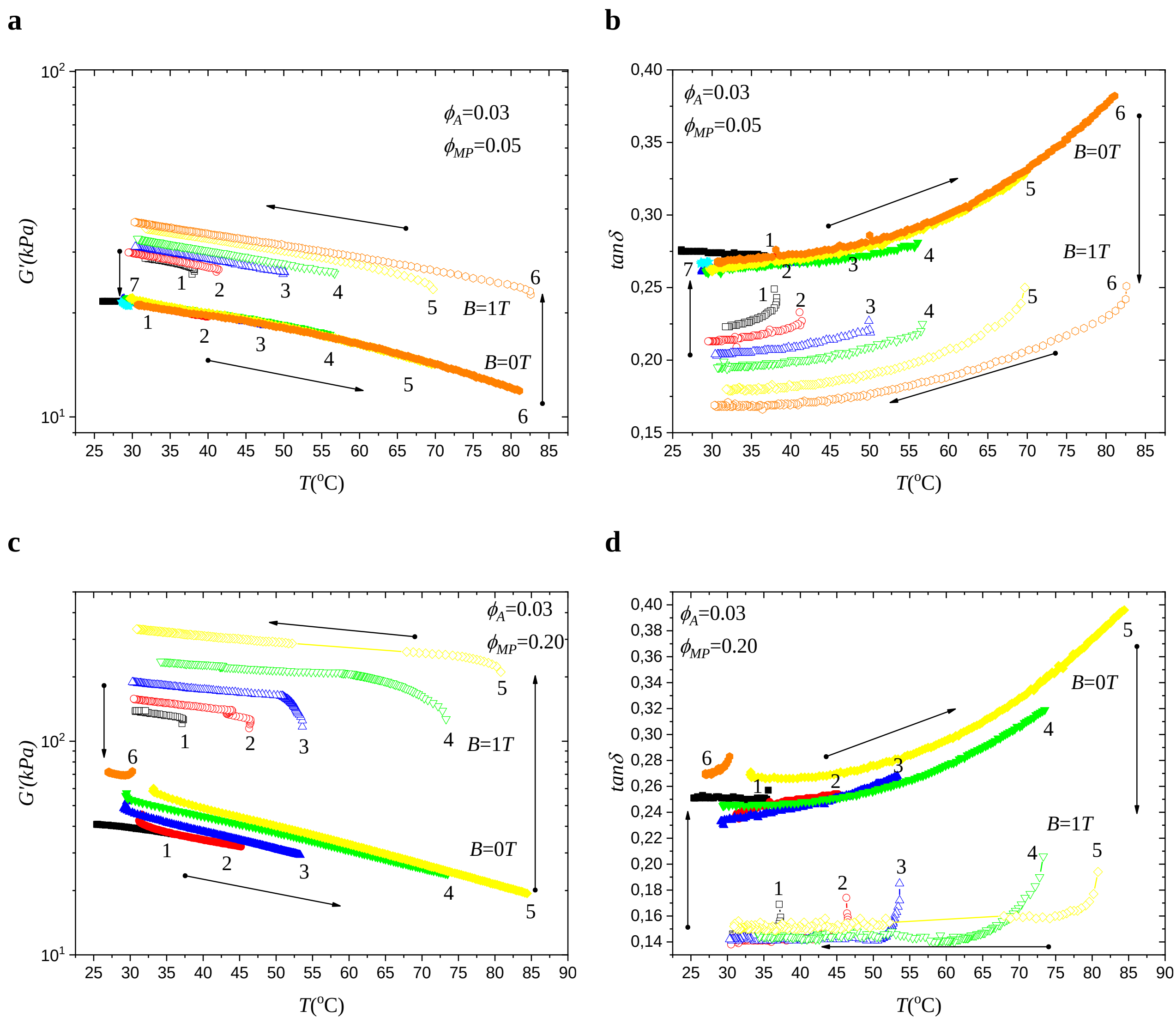

Figure S8. Creating anisotropic MP distribution in MATEs by using different treatment temperatures. Temperature dependence of (a,c) storage modulus and (b,d) damping factor for MATEs with (a,b) 5% MPs and (c,d) 20% MPs based on polymer matrix with 3% fraction of PS-block. Arrows show the direction of temperature change and digits near the curves indicate number of cycle.

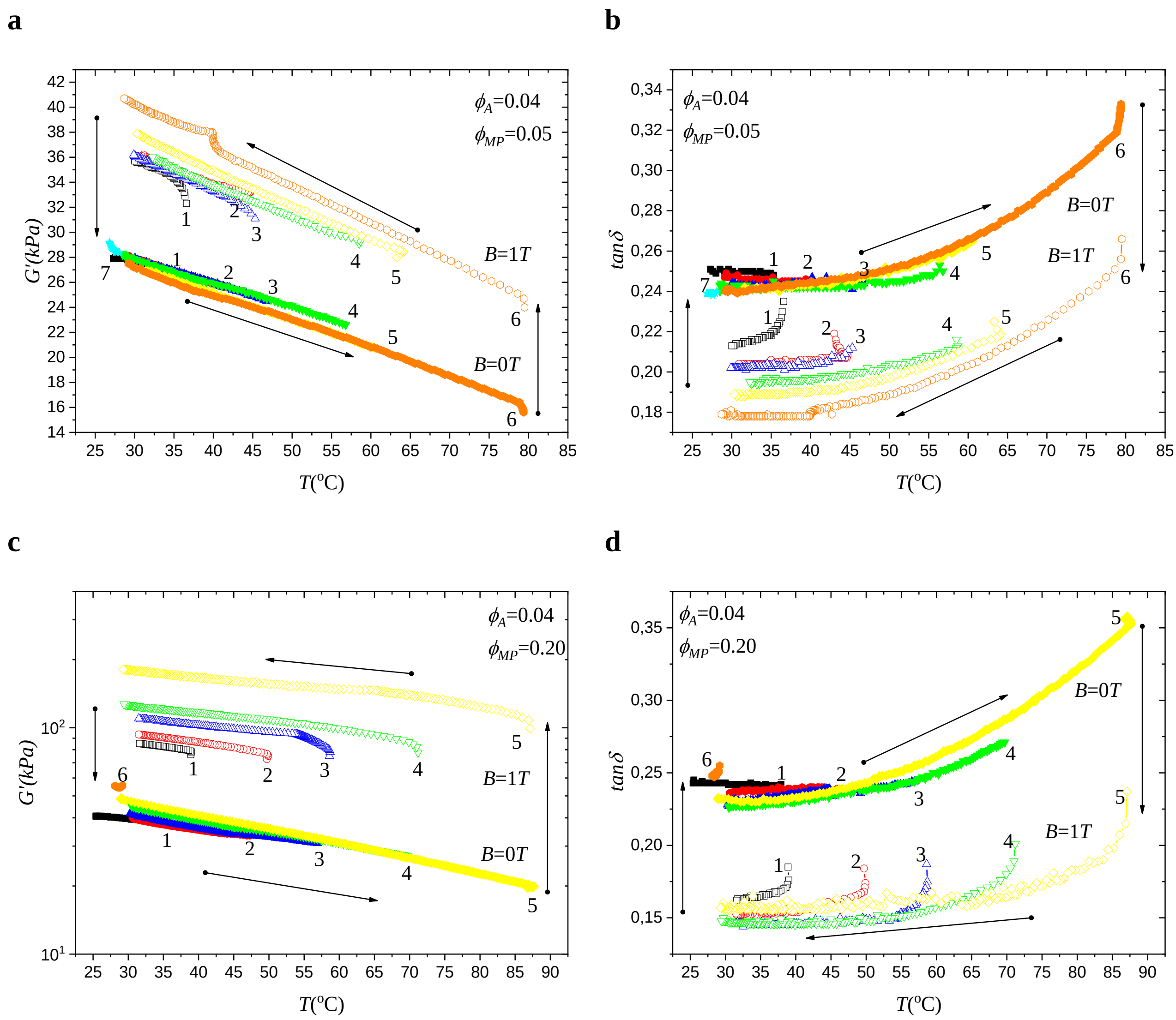

Figure S9. Creating anisotropic MP distribution in MATEs by using different treatment temperatures. Temperature dependence of (a,c) storage modulus and (b,d) damping factor for MATEs with (a,b) 5% MPs and (c,d) 20% MPs based on polymer matrix with 4% fraction of PS-block. Arrows show the direction of temperature change and digits near the curves indicate number of cycle.

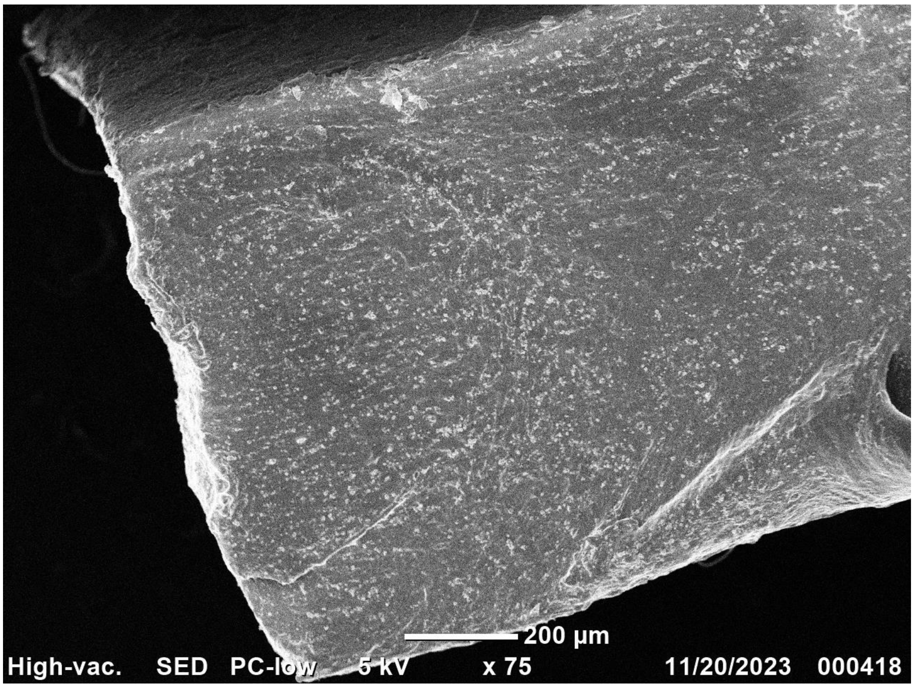

Figure S10. SEM image of the sample with 20% MPs based on polymer matrix with 3% fraction of PS-block after treatment in magnetic field parallel to the sample surface.
